# A Manual of Weights and Measures

**Published:** 1887-06

**Authors:** 


**A Manual of Weights and Measures** including Principles of Metrology, the Weights and Measures now in Use; Weight and Volume and their Reciprocal Relations, etc., with Rules and Tables. By Oscar Oldberg, Ph. D., Professor of Pharmacy, etc., Illinois College of Pharmacy. Second edition, revised. Chicago: Charles T. Johnson, 105 Madison st.

This is an improved edition of a valuable little work. Students of medicine, and especially pharmacists, should be thoroughly familiar with the various systems of weights and measures in use in all parts of the civilized world. This book is the most complete treatise on metrology to be had in convenient form, and we heartily recommend it.

